# Evaluation of two doses of etoricoxib, a COX-2 selective non-steroidal anti-inflammatory drug (NSAID), in the treatment of Rheumatoid Arthritis in a double-blind, randomized controlled trial

**DOI:** 10.1186/s12891-016-1170-0

**Published:** 2016-08-08

**Authors:** Kara Bickham, Alan J. Kivitz, Anish Mehta, Nancy Frontera, Sandhya Shah, Paul Stryszak, Zoran Popmihajlov, Paul M. Peloso

**Affiliations:** 1 Merck & Co., Inc, Kenilworth, NJ USA; 2 Altoona Center for Clinical Research, Duncansville, PA USA

## Abstract

**Background:**

Treatment with non-steroidal anti-inflammatory drugs (NSAID) is a common component of treatment regimens for rheumatoid arthritis (RA). Etoricoxib is a COX-2 selective NSAID that has demonstrated efficacy in the treatment of RA at a dose of 90 mg. The current study further evaluated the efficacy of etoricoxib 60 mg and 90 mg in RA patients with active disease.

**Methods:**

This was a 2-part, double-blind, placebo-controlled study in RA (NCT01208181). Patients were required to have a diagnosis of RA (according to ARA 1987 revised classification criteria) and were to demonstrate symptom flare upon discontinuation of previous NSAID treatment prior to randomization. Part I was a 6-week, placebo-controlled period to assess the efficacy of etoricoxib 90 mg and etoricoxib 60 mg, each compared to placebo, as well as to each other. Part II was a 6-week period to evaluate the potential benefit of dose escalation from etoricoxib 60 mg to etoricoxib 90 mg after 6 weeks exposure to etoricoxib 60 mg in Part I compared to maintaining a steady dose of etoricoxib 60 mg throughout Parts I and II. Primary endpoints were Disease Activity Score evaluating 28 joints and C reactive protein level (DAS28-CRP) index and Patient Global Assessment of Pain (Pain) score (0–100 mm VAS) after 6 weeks of treatment in Part I. Adverse events were monitored throughout the study.

**Results:**

In total, 1404 patients were randomized in a 2:7:7:8 ratio; 1228 patients completed Part I and 713 patients continued to Part II. Both etoricoxib doses were superior to placebo on both primary efficacy endpoints (*p* = 0.004 for 60 mg and *p* = 0.034 for 90 mg for DAS28-CRP; *p* < 0.001 for both doses for PGAP) in Part I. Further in Part I, etoricoxib 90 mg was not significantly different from 60 mg for DAS28-CRP, but did demonstrate a small, but statistically significant decrease in baseline PGAP score vs. 60 mg (*p* = 0.019). In Part II, there was no significant decrease in PGAP score after increasing to 90 mg in subjects with inadequate pain relief on 60 mg as compared to subjects who stayed on 60 mg. The incidence of AEs and SAEs were similar between etoricoxib 60 mg and 90 mg in both Part I and II.

**Conclusion:**

Both etoricoxib 90 mg and 60 mg are superior to placebo in relieving the symptoms of RA. Etoricoxib 90 mg vs 60 mg resulted in a statistically significant, though small, improvement in PGAP score, but not DAS28-CRP. Dose escalation from 60 mg to 90 mg in pain inadequate responders did not significantly improve efficacy. These results confirm the efficacy and tolerability of etoricoxib 90mg in patients with RA. In addition, this study demonstrated that etoricoxib 60 mg is also efficacious and well-tolerated in RA.

**Clinical Trial Registration:**

NCT01208181 (registered September 22, 2010).

**Electronic supplementary material:**

The online version of this article (doi:10.1186/s12891-016-1170-0) contains supplementary material, which is available to authorized users.

## Background

Rheumatoid arthritis (RA) is a disabling condition associated with symptoms that ultimately lead to significant disability. Among these symptoms, pain is a primary complaint according to patients that most often leads them to seek treatment [1]. Pain in chronic conditions can have a significant impact on the lives of patients and often includes interference in daily life and the reduction of the ability of patients to work and engage in social activities, thus affecting psychological wellbeing [2].

Biologic treatments targeting the immunological pathology of RA have greatly improved patient outcomes and have made remission and prevention of joint destruction possible [3, 4]. However, despite advances in treatment with biologics, research has suggested that RA patients can continue to experience significant levels of pain even when underlying disease markers of RA (i.e. DAS-28) are controlled by modern therapeutic regimens [5, 6]. Nonsteroidal anti-inflammatory drugs (NSAIDs) are treatments that target the cyclooxygenase (COX) 1 and 2 isozymes and are often recommended as first-line therapy in rheumatic joint disorders [4]. NSAIDs provide pain relief, which can greatly improve quality of life, and may continue to be important in those patients who continue to possess high levels of pain from RA despite adequate disease modifying treatment [1, 7].

Etoricoxib is a COX-2 selective NSAID with high selectivity for COX-2 vs. COX-1 [8] that has demonstrated efficacy in the treatment of pain from RA. Clinical trial data suggest that NSAIDs such as etoricoxib can provide symptomatic benefits to RA patients, complimenting the disease modifying effects observed with biologic therapy [9]. Previous studies have evaluated several doses of etoricoxib identifying 90 mg as the optimal dose, although the 60 mg dose has also demonstrated symptomatic improvements as compared with placebo, particularly for pain [10–12].

Because previous studies suggest that 60 mg can provide significant pain improvement and therefore has some clinically meaningful effect, this study was done to specifically evaluate the effect of etoricoxib 60 mg and 90 mg vs. placebo as well as compare both doses to each other in RA. The potential benefit of dose escalation to 90 mg in patients who do not respond to treatment with 60 mg was also evaluated. Guidelines recommend the use of the lowest efficacious dose of NSAIDs to provide symptomatic treatment while mitigating potential adverse experiences (AEs). Data from this study helps to further define an additional efficacious dose of etoricoxib for RA patients, leading to greater dosing flexibility and an optimized benefit/risk ratio for individual patients receiving etoricoxib.

## Methods

This study (Clinical Trials Registry # NCT01208181, Sponsor Protocol MK-0663 Protocol 107) was conducted at 211 study centers in Argentina, Austria, Canada, Columbia, Czech Republic, Finland, Germany, Guatemala, India, Lithuania, Mexico, Panama, Peru, Poland, Romania, Russia, Slovakia, South Africa, Taiwan, the United Kingdom, and the United States. The study was initiated in September 2010 and completed in August 2014. The protocol for the study was approved by local institutional review boards or ethical review committees (Additional file 1) and was conducted according to principles of Good Clinical Practice. Patients provided informed consent prior to participation in the study.

### Patients

Male and female patients, at least 18 years of age, with a diagnosis of RA made at least 6 months prior to the screening visit, who met American Rheumatology Association (ARA) 1987 revised classification criteria, who demonstrated a prior clinical response to NSAIDs and who took NSAIDs on a regular basis were enrolled. Patients on stable doses of selected non-study anti-rheumatic therapies participated in the study (and remained on their non-study anti-rheumatic therapy throughout) provided that activity and flare criteria were met following NSAID washout. Following discontinuation (“washout”) of NSAIDs, patients must have demonstrated sufficient disease activity defined by activity criteria and worsening in signs and symptoms from the screening visit (Visit 1). Flare criteria included all three of the following: 1) ≥6 tender joints and an increase in number of tender joints of at least 20 % (or a minimum of 2 tender joints, whichever is greater) compared to values prior to washout; 2) ≥3 swollen joints and an increase in number of swollen joints of at least 20 % (or a minimum of 2 swollen joints, whichever is greater) compared to the values prior to washout; and 3) Investigator’s Global Assessment of Disease Activity must be “fair”, poor”, or “very poor post washout”. One of the following two criteria were also to be met in order for patients to be randomized to study treatment: 4) Duration of morning stiffness ≥45 min, and an increase in duration of morning stiffness of at least 15 min compared to results prior to washout; OR 5) Patient’s assessment of pain >40 mm and an increase of at least 10 mm in patient’s assessment of pain over the value prior to washout.

### Study design

**Fig. 1:**
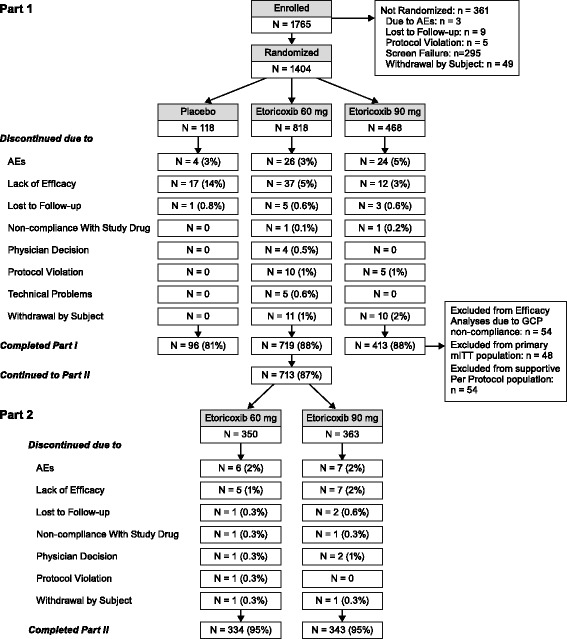
CONSORT diagram/study design

This was a 2-part, double-blind, 12-week placebo-controlled study in patients with RA. Eligible patients were randomized at Visit 2 (randomization visit) to 1 of the 4 treatment sequences (treatment in Part I/treatment in Part II) in a 2:7:7:8 ratio: Treatment Sequence 1 (*N* = 118): placebo/patient completed the study at the end of Part I; Treatment Sequence 2 (*N* = 409): etoricoxib 60 mg in Part I/etoricoxib 60 mg in Part II; Treatment Sequence 3 (*N* = 409): etoricoxib 60 mg in Part I/etoricoxib 90 mg in Part II; Treatment Sequence 4 (*N* = 468): etoricoxib 90 mg/patient completed the study at the end of Part I (Fig. 1). Randomization was stratified by concomitant use of biological disease modifying therapy (biologics) and the proportion of concomitant biologics users was capped at 50 % of the total study population.

**Fig. 2:**
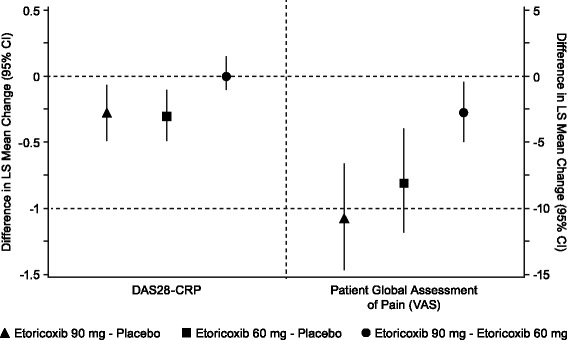
Co-primary endpoints (mITT population). 'Patient Global Assessment of Pain was measured on a 0- to 100-mm VAS, with a lower value representing a better response. DAS28-CRP ranges from 0 to 10, with lower scores representing a better response. LS = Least-Squares; CI = Confidence Interval

### Efficacy parameters

The primary hypothesis was that etoricoxib 90 mg and etoricoxib 60 mg will demonstrate superior clinical efficacy compared with placebo after 6 weeks of treatment (end of Part I), as assessed by the co-primary endpoints.

The co-primary endpoints included: 1) the time-weighted average change from baseline in the Disease Activity Score evaluating 28 joints using C reactive protein (DAS28-CRP) in Part I (etoricoxib 90 mg vs. placebo and etoricoxib 60 mg vs. placebo); and 2) time-weighted average change from baseline in Patient Global Assessment of Pain (Pain) in Part I (etoricoxib 90 mg vs. placebo and etoricoxib 60 mg vs. placebo). This study was to be declared positive if etoricoxib 90 mg is shown to be superior to placebo in both the DAS28-CRP and Pain (VAS).

The key secondary endpoints were time-weighted average change from baseline in DAS28-CRP and Pain in Part I (etoricoxib 90 mg vs. etoricoxib 60 mg), and change from Week 6 to Week 10 and 12 in Pain (inadequate responders to etoricoxib 60 mg in Part I and received etoricoxib 90 mg in Part II vs. inadequate responders to etoricoxib 60 mg in Part I and remained on etoricoxib 60 mg in Part II). Patients who showed less than 50 % improvement in Pain (VAS) from baseline at Weeks 6 were to be considered as inadequate pain responders.

Other secondary endpoints included the proportion of American College of Rheumatology Responder Index criteria (20 %) (ACR20) responders in Part I, the proportion of patients who discontinued due to a lack of efficacy, time-weighted average response in Patient Global Assessment of Response to Therapy (PGART) (Likert 0 to 4) in Part I, and the average change from Week 6 in PGART (Likert 0 to 4) over Weeks 10 and 12.

### Safety parameters

Safety assessments included physical examinations, vital signs, and some hematology and chemistry tests (i.e., hemoglobin, hematocrit, alanine aminotransferase [ALT], aspartate transaminase [AST], and creatinine), urine and serum ß-human chorionic gonadotropin (ß-hCG) for women of childbearing potential, and spontaneous AE reporting (up to 28 days [+2 days] after the last dose of study medication). All thrombotic CV serious adverse experiences (SAE) and upper GI events (such as perforation, ulcer, or upper GI bleeding) that occurred in patients during the study or within 28 days of its use were subject to adjudication by a committee external to the Sponsor. Adjudication was performed during the trial by an independent panel of experts in cardiology, neurology, gastroenterology, and peripheral vascular disease, who remained blinded to therapies.

### Statistical planning and analysis

The modified intention-to-treat (mITT; consisting of all patients who received ≥1 dose of study medication, had ≥1 post-randomization measurement, and had baseline data) population served as the primary population for the primary analysis as well as key secondary endpoints. The sample size was planned to provide between 81 and 98 % power depending on the endpoint or comparison. The 2 primary endpoints were analyzed using analysis of covariance (ANCOVA). For each primary endpoint, the difference between each of the etoricoxib doses (90 mg and 60 mg) from placebo (dose-response effect) was assessed by Tukey-Ciminera-Heyse trend test based on the least-squares (LS) means derived from this ANCOVA (2-sided, α = 0.05). An etoricoxib dose was declared superior to placebo if it demonstrated significantly (*p* ≤ 0.05) greater efficacy than placebo on both of these endpoints. For the key secondary endpoints, Hochberg’s procedure was applied for determining the significance of treatment differences (etoricoxib 90 mg vs. etoricoxib 60 mg) for these 2 endpoints starting with α = 0.05, (2-sided).

Patients with less than 50 % improvement in Pain (VAS) score from baseline to Week 6 are defined as inadequate responders. For the average change from Week 6 in Pain (VAS) over Weeks 10 and 12, among pain inadequate responders in Part I, the difference between the etoricoxib 60 mg/90 mg and etoricoxib 60 mg/60 mg treatment sequences were compared using an ANCOVA. The benefit of etoricoxib dose increase from 60 mg (in Part I) to 90 mg (in Part II) was indicated if the nominal p-value (without multiplicity adjustment) for the difference in LS means was ≤0.20 (2-sided) in favor of the 90 mg dose (alpha level agreed to in consultations with EU regulatory authorities during the planning of the study). To control the experiment-wise Type I error rate in Part I of the study, the primary endpoints and the first key secondary endpoint in Part I were tested sequentially. No multiplicity adjustments were made for this key secondary endpoint.

To evaluate the consistency of treatment effects, post-hoc analyses were performed separately for subgroups defined by ≤ or > median values for Pain (VAS), DAS28-CRP, Tender Joint Count (68 joints), and Swollen joint Count (66 joints).

For pre-specified AEs of interest, *p*-values and/or 95 % CI’s were provided using the Miettinen and Nurminen method for treatment group comparisons. All other AEs were summarized with counts and percentages.

## Results

### Patients

Of the 1404 patients who were randomized, 83.5 % were female and the mean age was 53.8 years. The mean duration of rheumatoid arthritis (RA) was 5.6 years and ranged from less than 1 year to 36 years. At baseline, 235, 925, and 244 patients were American Rheumatology Association (ARA) Functional Class I, II, or III, respectively. There were no clinically meaningful differences between the treatment groups for these or any other baseline disease-related characteristics (Table 1). In Part I, 118, 818, and 468 patients received placebo, etoricoxib 60 mg, and etoricoxib 90 mg, respectively. There were 1228 patients who completed Part I (87 %) with the most common reasons for discontinuation due to lack of efficacy and adverse experiences (Fig. 1). There were 713 patients who entered Part II with 350 continuing on 60 mg and 363 switched to etoricoxib 90 mg; 677 of these patients completed Part II (95 %).

**Table 1:** Baseline demographics

	Placebo *N* = 118	Etoricoxib 60 mg *N* = 818	Etoricoxib 90 mg *N* = 468	Total *N* = 1404
	n (%)	n (%)	n (%)	n (%)
Gender				
Female	100 (84.7)	677 (82.8)	395 (84.4)	1172 (83.5)
Age (Years)				
Mean (SD)	53.6 (11.0)	53.8 (11.9)	54.0 (12.3)	53.8 (12.0)
Min-Max Age	27 – 80	18 – 83	19 – 84	18 – 84
Race				
White	85 (72.0)	626 (76.5)	348 (74.4)	1059 (75.4)
Asian	16 (13.6)	93 (11.4)	55 (11.8)	164 (11.7)
Multi-racial	10 (8.5)	74 (9.0)	42 (9.0)	126 (9.0)
Black	4 (3.4)	17 (2.1)	12 (2.6)	33 (2.4)
Other	3 (2.5)	8 (1.0)	11 (2.4)	22 (1.6)
Duration of RA (Years)				
Mean (SD)	4.2 (4.3)	5.7 (6.7)	5.8 (6.5)	5.6 (6.5)
ARA Functional Class				
Class I	18 (15.3)	138 (16.9)	79 (16.9)	235 (16.7)
Class II	76 (64.4)	535 (65.4)	314 (67.1)	925 (65.9)
Class III	24 (20.3)	145 (17.7)	75 (16.0)	244 (17.4)
Biologics User	16 (13.6)	113 (13.8)	64 (13.7)	193 (13.7)
Methotrexate User	43 (36.4)	285 (34.8)	170 (36.3)	498 (35.5)
Low-dose Corticosteroid User	25 (21.2)	208 (25.4)	126 (26.9)	359 (25.6)
DMARD User	56 (47.5)	363 (44.4)	219 (46.8)	638 (45.4)
DMARD or Corticosteroid	61 (51.7)	425 (52.0)	247 (52.8)	733 (52.2)

### Efficacy

#### Etoricoxib 60 mg and 90 mg vs. placebo

Improvements in the primary endpoint of time-weighted average change from baseline DAS28-CRP score were significantly greater in the etoricoxib 90 mg and etoricoxib 60 mg groups compared with the placebo group (*p* = 0.034 and *p* = 0.004 vs. placebo, respectively) over 6 weeks of treatment in Part I in patients included in the primary mITT population (Fig. 2).

Improvements in the primary endpoint of time-weighted average change from baseline Pain score were significantly greater in the etoricoxib 90 mg and etoricoxib 60 mg groups compared with the placebo group (*p* < 0.001 for both treatments vs. placebo) over 6 weeks of treatment in Part I. The treatment difference from placebo for etoricoxib 90 mg, but not 60 mg, met the −10 mm MCID pre-specified in the protocol (Fig. 2).

Results for secondary endpoints are provided in Table 2 and 3. The proportions of ACR20 responders in the etoricoxib 90 mg and etoricoxib 60 mg groups were greater than those in the placebo group (nominal *p* < 0.001 and nominal *p* = 0.017, respectively). The time-weighted average response PGART score in the etoricoxib 90 mg and etoricoxib 60 mg groups was greater than that for the placebo group (nominal *p* < 0.001 and nominal *p* < 0.001, respectively) over 6 weeks of treatment in Part I. The etoricoxib 90 mg and etoricoxib 60 mg groups had smaller proportions of patients who discontinued due to lack of efficacy compared with the placebo group ; 2.3 % of etoricoxib 90 mg patients and 3.8 % of etoricoxib 60 mg patients discontinued due to lack of efficacy vs. 13.5 % of placebo patients (nominal *p* < 0.001 and nominal *p* < 0.001, respectively). Results for additional endpoints such as Tender Joint Counts, Swollen Joint Counts, Patient and Investigator Assessments of Disease Activity, Health Assessment Questionnaire, and C-Reactive Protein were generally similar to results for the primary and secondary endpoints (Additional file 2: Table S1).

**Table 2:** Summary of primary and secondary efficacy endpoints during part I (6 weeks of treatment)

	Placebo	Etoricoxib 60 mg	LS Mean Difference vs. Placebo; *p*-value^a,b^	Etoricoxib 90 mg	LS Mean Difference vs. Placebo; *p*-value	LS Mean Difference Between 60 mg and 90 mg; *p*-value^a,b^
Co-Primary Endpoints^a^						
LS mean change from baseline DAS28-CRP (95 % CI)	- 1.10 (-1.29, -0.90)	- 1.39(-1.48, -1.30)	-0.29 (-0.49, -0.09); *p* = 0.004	- 1.37 (-1.48, -1.26)	-0.27 (-0.48, -0.06); *p* = 0.034	0.02 (-0.10, 0.14); *p* = 0.730
LS mean change from baseline in Pain - PGAP (95 % CI)	- 20.26 (-24.04, -16.48)	- 28 .25 (-30.05, -26.44)	-7.99 (-11.85, -4.13); *p* < 0.001	- 30.96 (-33.13, -28.79)	-10.70 (-14.74, -6.66); *p* < 0.001	-2.71 (-4.98, -0.45); *p* = 0.019
Secondary Endpoints^b^						
Proportions of Patients Who Met ACR20 (%)	41/111 (36.94)	377/769 (49.02)	12.09 (2.52, 21.66); *p* = 0.017	242/440 (55.00)	18.19 (8.17, 28.22); *p* < 0.001	6.03 (0.22, 11.85); *p* = 0.043
Time-weighted Average (LS Mean) Response over 6 Weeks in PGART (95 % CI)	2.00 (1.83, 2.18)	2.46 (2.38, 2.55)	0.46 (0.28, 0.64); *p* < 0.001	2.48 (2.38, 2.58)	0.47 (0.29, 0.66); *p* < 0.001	0.01 (-0.09, 0.12); *p* = 0.815
Proportion of Patients Who Discontinued due to Lack of Efficacy (%)	15/111 (13.51)	29/769 (3.77)	-9.74 (-16.24, -3.24); *p* < 0.001	10/440 (2.27)	-11.24 (-17.75, -4.73); *p* < 0.001	-1.50 (-3.44, 0.44); *p* = 0.178

^a^ *p*-values for primary endpoints were adjusted for multiplicity; statistical significance is achieved if *p* ≤ 0.05

^b^ *p*-values for secondary endpoints were not adjusted for multiplicity and are therefore nominal; statistical significance cannot be inferred from p-values for these endpoints

**Table 3:** Summary of secondary endpoints during part II among inadequate responders from part I

	Etoricoxib 60 mg (Part I)/60 mg (Part II)	Etoricoxib 60 mg (Part I)/90 mg (Part II)	LS Mean Difference Between Etoricoxib 60 mg/90 mg vs. Etoricoxib 60 mg/60 mg; *p*-value^a^
LS Mean Change from Week 6 in PGAP over Weeks 10 and 12	−11.96 (−14.96, −8.97)	−10.35 (−13.32, −7.39)	1.61 (−0.49, 3.71); *p* = 0.327
LS Mean Change from Week 6 in PGART over Weeks 10 and 12	0.33 (0.19, 0.46)	0.24 (0.10, 0.37)	−0.09 (−0.18, 0.00); *p* = 0.215
LS Mean Change from Week 6 in DAS28-CRP over Weeks 10 and 12	−0.35 (−0.51, −0.18)	−0.39 (−0.55, −0.23)	−0.05 (−0.22, 0.13); *p* = 0.611

^a^ *p*-values for secondary endpoints were not adjusted for multiplicity and are therefore nominal; statistical significance cannot be inferred from *p*-values for these endpoints

#### Etoricoxib 60 mg vs. Etoricoxib 90 mg

For the comparison of etoricoxib 60 mg and 90 mg, the results of hypothesis testing showed no statistically significant difference between the etoricoxib 90 mg group and the etoricoxib 60 mg group in the DAS28-CRP co-primary endpoint (*p* = 0.730). For the co-primary endpoint of Pain, etoricoxib 90 mg led to statistically significantly lower Pain scores compared with etoricoxib 60 mg (*p* = 0.019). For the secondary endpoint of the proportion of ACR20 responders, the etoricoxib 90 mg group had a greater proportion of responders than that in the etoricoxib 60 mg group (nominal *p* = 0.043). There was no difference between the etoricoxib 90 mg group and the etoricoxib 60 mg group in the time-weighted average response PGART score. The proportion of patients who discontinued due to lack of efficacy in the etoricoxib 60 mg group and the 90 mg group was similar (Table 2).

In Part II, there was no evidence of incremental benefit with increasing the dose from 60 mg to 90 mg among inadequate pain responders who started on etoricoxib 60 mg in Part I and increased to etoricoxib 90 mg in Part II, versus remaining on etoricoxib 60 mg, according to the time-weighted change in Pain, PGART, and DAS28-CRP. Results for other, tertiary endpoints such as Tender Joint Counts, Swollen Joint Counts, Patient and Investigator Assessments of Disease Activity, Health Assessment Questionnaire, and C-Reactive Protein were generally similar to results for Pain, PGART, and DASE28-CRP (Additional file 2: Table S2).

#### Post-hoc analyses of baseline subgroups

**Fig. 3:**
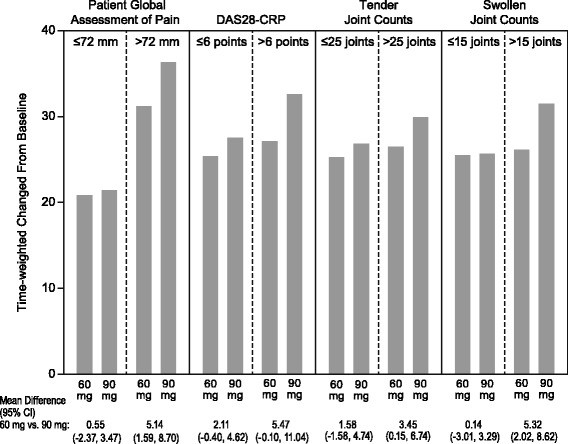
Treatment differences by baseline median subgroups (60 mg vs. 90 mg) for time-weighted change from baseline in efficacy endpoints over 6 weeks in Part I

Across all four endpoints (i.e., Pain, DAS28-CRP, Tender Joint Counts, and Swollen Joint Counts), greater improvement was observed with etoricoxib treatment vs. placebo in those subjects with higher baseline scores when compared with those subjects with lower baseline scores (cutoffs based on median baseline values). When comparing the two doses, the 90 mg dose demonstrated greater improvement of scores compared with the 60 mg dose in subjects who had higher baseline scores (Fig. 3).

### Safety and tolerability

In Part I, AEs occurred in 30 (25 %), 248 (30 %), and 168 (36 %) patients in the placebo, etoricoxib 60 mg, and etoricoxib 90 mg groups, respectively. There were 9 patients with serious AEs: 7 (0.9 %) in the etoricoxib 60 mg group and 2 (0.4 %) in the etoricoxib 90 mg group. There were no deaths in this study. Additionally, there were 55 patients who discontinued due to clinical AEs: 4 (3.4 %), 27 (3.3 %), and 24 (5.1 %) in the placebo, etoricoxib 60 mg, and etoricoxib 90 mg groups, respectively. The most commonly reported AE was headache (3.1 to 5.1 % of patients) (Table 4).

**Table 4:** Summary of AEs during Part I

	Placebo *n* = 118	Etoricoxib 60 mg *n* = 819	Etoricoxib 90 mg *n* = 467
N (%) with AEs	30 (25.4)	248 (30.3)	168 (36.0)
N (%) with AEs determined by the investigator to be drug related	8 (6.8)	64 (7.8)	59 (12.4)
N (%) with serious AEs	0 (0.0)	7 (0.9)	2 (0.4)
N (%) who discontinued due to AEs	4 (3.4)	27 (3.3)	24 (5.1)
Most Common AEs (incidence >2 % in one or more treatment groups)			
Upper abdominal pain	2 (1.7)	12 (1.5)	11 (2.4)
Nausea	0 (0.0)	19 (2.3)	5 (1.1)
Peripheral edema	0 (0.0)	8 (1.0)	10 (2.1)
Nasopharyngitis	1 (0.8)	14 (1.7)	14 (3.0)
Blood pressure increased	2 (1.7)	10 (1.2)	13 (2.8)
Headache	6 (5.1)	25 (3.1)	17 (3.6)
Hypertension	0 (0.0)	20 (2.4)	15 (3.2)
Prespecified AEs of Interest			
Hypertension-related AEs^a^	2 (1.7)	34 (4.2)	26 (5.6)
Discontinuation due to hypertension-related AEs	2 (1.7)	3 (0.4)	11(2.4)
Edema-related AEs^b^	0 (0.0)	8 (1.0)	10 (2.1)
Discontinuation due to edema-related AEs	0 (0.0)	0 (0.0)	2 (0.4)
Congestive heart failure, pulmonary edema, or cardiac failure	0 (0.0)	0 (0.0)	0 (0.0)

^a^Hypertension-related AEs included development of hypertension or worsening of preexisting hypertension

^b^Edema-related AEs included edema, lower extremity edema, and peripheral edema

In Part II, there were 67 (19 %) and 89 (25 %) patients with AEs in the group of patients who continued on etoricoxib 60 mg in Part II and the group who received etoricoxib 90 mg in Part II. There were 9 patients with serious AEs: 4 (1.1 %) on etoricoxib 60 mg and 5 (1.4 %) on etoricoxib 90 mg). Discontinuations due to AEs occurred in 5 (1.4 %) patients on etoricoxib 60 mg and 7 (1.9 %) patients on etoricoxib 90 mg. The most commonly reported AE was nasopharyngitis (2.3 to 2.5 % of patients) (Table 5).

**Table 5:** Summary of AEs during Part II

	Etoricoxib 60 mg/60 mg *n* = 350	Etoricoxib 60 mg/90 mg *n* = 363
N (%) with AEs	67 (19.1)	89 (24.5)
N (%) with AEs determined by the investigator to be drug related	15 (4.3)	16 (4.4)
N (%) with serious AEs	4 (1.1)	5 (1.4)
N (%) who discontinued due to AEs	5 (1.4)	7 (1.9)
Most Common AEs (incidence >2 % in one or more treatment groups)		
Nasopharyngitis	8 (2.3)	9 (2.5)
Prespecified AEs of Interest		
Hypertension-related AEs	8 (2.3)	7 (1.9)
Discontinuation due to hypertension-related AEs	0 (0.0)	0 (0.0)
Edema-related AEs	1 (0.3)	2 (0.6)
Discontinuation due to edema-related AEs	0 (0.0)	0 (0.0)
Congestive heart failure, pulmonary edema, or cardiac failure	0 (0.0)	0 (0.0)

Prespecified AEs of interest (i.e., gastrointestinal or renovascular AEs) were similar among the treatment groups in both Part I and Part II. There were 2 cases of confirmed/adjudicated thromboembolic CV events during the study (both in the etoricoxib 60 mg Part I/90 mg Part II group during Part II of the study). These included an acute myocardial infarction and an ischemic stroke due to cardiac thrombus. There were 3 cases of confirmed/adjudicated GI events during the study (all in the etoricoxib 60 mg group during Part I). None of the events had an adjudication outcome of confirmed upper GI bleed.

## Discussion

This study was done in order to further explore dosing options for patients with RA who respond to etoricoxib. In order to minimize the risk of AEs, the use of the lowest dose of an NSAID, such as etoricoxib, that achieves adequate symptomatic control of RA symptoms is recommended. This is particularly important in a population, such as RA patients, who may be on multiple medications and need treatment for chronic pain. In a previously reported dose ranging study, etoricoxib 90 mg demonstrated statistically significant improvement vs. placebo in the primary endpoint of the proportion of patients achieving an ACR20 response, whereas etoricoxib 60 mg only demonstrated a numerical improvement that approached statistical significance [12]. However, both the 90 mg and 60 mg doses of etoricoxib provided statistically significant improvement for the treatment of pain. The current trial was conducted, therefore, to further explore the treatment effect of the 60 mg and 90 mg dose in RA patients with active disease.

In this study, both etoricoxib 60 mg and 90 mg were superior to placebo for both the co-primary endpoints of DAS28-CRP and PGAP. These results confirm previously reported studies that show efficacy of 90 mg in the treatment of RA and the previous dose ranging study that showed that 60 mg also provided significant improvement in the treatment of pain vs. placebo in patients with RA [11, 12]. When compared with each other, there was no significant difference between the etoricoxib 90 mg dose and 60 mg dose for the co-primary endpoint of DAS28-CRP. For the other co-primary endpoint of PGAP that specifically evaluated pain, etoricoxib 90 mg was statistically significantly superior to etoricoxib 60 mg. However, in patients who had an inadequate response to treatment with etoricoxib 60 mg after 6 weeks, switching to the 90 mg dose did not significantly improve the pain scores.

The use of NSAIDs such as etoricoxib remains an important part of the treatment of RA. Despite widespread adoption of biologic DMARDs with their ability to halt disease progression and provide radiographic improvement in the last decade, the use of agents that control pain has persisted [5, 6]. A previous analysis demonstrated that etoricoxib provided important pain relief in patients with RA who continued to have pain despite biologic DMARD treatment and/or treatment with corticosteroids [9]. Therefore pain relief in RA remains an additional important treatment goal in spite of advances in disease modifying therapy.

These results show that the etoricoxib 60 mg dose is an effective and viable option in the treatment of RA and can improve pain from RA and DAS28-CRP scores while minimizing the drug dose. The variability in patient pain responses is highlighted in post-hoc analyses that evaluated patients with greater baseline severity vs. those with lower baseline severity. In patients with higher baseline scores, there was a greater numerical degree of improvement and a larger difference in effect between the 60 mg and 90 mg doses, particularly for the Pain endpoint. Previous research in acute pain models have demonstrated that the evaluation of patients who experience greater levels of pain can better differentiate the efficacy of analgesic medications [13]. These data show that the availability of multiple doses of etoricoxib may be advantageous for patients who experience severe RA symptoms and who respond to etoricoxib in a dose-dependent manner. Greater improvements in patients with greater baseline symptom severity were seen in the endpoint evaluating pain as well as DAS28-CRP, Swollen Joint Counts, and Tender Joint Counts.

There have recently been efforts to simplify the quantification of efficacy through the identification of minimally clinically important differences [14]. In this study, we prespecified a −0.5 unit difference from placebo in DAS28-CRP and a −10 mm difference from placebo in Pain. The −0.5 unit difference from placebo for DAS28-CRP is based on previous research with biologic agents [15]. Whether or not this benchmark applies to treatment with NSAIDs is uncertain, but was used as a starting point in this study. The MCID of −10 mm difference from placebo for Pain was established based on the work of the Initiative on Methods Measurement and Pain Assessment in Clinical Studies (IMMPACT), which suggested that a 10 % change from baseline with regard to pain scores represented the lower boundary of clinical importance [16]. However, since the development of an MCID for pain was based on separating active treatment from placebo and not separating active treatment arms, it is possible that patients experience meaningful clinical improvement at the 90 mg vs. 60 mg dose at a smaller difference in pain score than that specified by the MCID. The assignment of MCIDs for pain relief in RA patients is also problematic because literature is scarce for RA specifically. In this study, etoricoxib did not reach −0.5 unit difference from placebo for the DAS28-CRP endpoint. Etoricoxib 90 mg, but not etoricoxib 60 mg, reached the −10 mm difference for Pain.

The primary endpoint, DAS28-CRP, was developed to measure effectiveness for DMARDs and is therefore commonly used in RA trials, however it may not be the best tool for evaluating NSAID efficacy because NSAIDs in general do not have an effect on CRP in RA patients [17]. While NSAIDs may show an effect on tender joint counts, DAS28-CRP results may be blunted due to a lack of effect on CRP, leading to smaller treatment effects.

NSAIDs have been associated with safety and tolerability risks, particularly gastrointestinal, renovascular, and cardiovascular risks, which necessitates an individualized approach to treat pain with these medications [18–20]. This study did not identify new AEs and was consistent with previous research on etoricoxib in the treatment of RA. The etoricoxib groups had a low proportion of patients with serious AEs while the placebo group had no serious AEs. There were dose-related incidences of hypertension-related AEs with the largest proportion of patients with these AEs occurring in the 90 mg group. Renovascular AEs such as hypertension have previously been associated with etoricoxib [21].

Previous studies have identified a risk of thrombotic CV events and GI ulcers and bleeding with the use of NSAIDs [20, 22]. These events are rare and require large outcome studies to properly identify to assess the risk of an individual NSAID. The CV risk of etoricoxib, a COX-2 selective NSAID, was evaluated compared with the traditional NSAID, diclofenac, in a large outcome trial; both medications were found to have a similar CV risk profile. Additionally, the GI profile of etoricoxib and diclofenac were compared and demonstrated that etoricoxib was associated with fewer uncomplicated upper GI events vs. diclofenac, but a similar incidence of complicated events. CV and GI events were rare with a similar incidence between treatment groups in this study.

This study was limited in that it was not designed nor powered to properly evaluate rare AEs of interest for NSAIDs such as CV and GI AEs. Additionally, this trial enrolled patients who previously demonstrated a response to NSAID treatment and who exhibited a flare of symptoms upon withdrawal of previous NSAID therapy. Our study included NSAID responders and used flare criteria in order to address specific objectives regarding potential differences in efficacy between to different doses of etoricoxib. This is a common design element in NSAID trials and recent research has shown that flare designs do not affect the treatment effect sizes observed in clinical trials compared with non-flare designs [23].

## Conclusion

In summary, in this study, both etoricoxib 90 mg and 60 mg once daily were superior to placebo in relieving the signs and symptoms of RA. Furthermore, etoricoxib 90 mg once daily compared with 60 mg once daily resulted in a statistically significant, though small, improvement in Patient Global Assessment of Pain (PGAP), but not Disease Activity Score (DAS28-CRP). Inadequate pain responders on etoricoxib 60 mg did not benefit from dose escalation to etoricoxib 90 mg. Both etoricoxib 90 mg and 60 mg were well tolerated confirming that etoricoxib 90 mg and 60 mg once daily are safe and effective treatment regimens for relieving the signs and symptoms of RA. Importantly, these data provide evidence that etoricoxib 60 mg is an additional efficacious etoricoxib dose that can provide clinicians with increased dosing flexibility to treat individual patient needs. The sum of the evidence suggests that for most patients with RA, the minimum clinically effective dose of etoricoxib is 60 mg once daily. However, in patients with higher baseline pain, etoricoxib 90 mg may provide better symptom control than 60 mg. A range of effective doses (60 or 90 mg) has now been described, which will allow for more individualized treatment recommendations by healthcare providers, consistent with dosing regimens of other NSAIDs used in the management of RA.

## Abbreviations

AE, adverse experience; ANCOVA, analysis of covariance; COX, cyclooxygenase; DAS, disease activity score; NSAID, nonsteroidal anti-inflammatory drug; PGAP, patient global assessment of pain; RA, rheumatoid arthritis; SAE, serious adverse experience

## Additional files

Additional file 1: List of Ethics Committees. (DOCX 38 kb) Additional file 2: Table S1. Summary of Additional Endpoints during Part I (6 weeks of treatment). **Table S2.** Summary of Additional Endpoints during Part II Among Inadequate Responders from Part I. (DOCX 21 kb)
