# Supplementary material for: Evaluation of two doses of etoricoxib, a COX-2 selective non-steroidal anti-inflammatory drug (NSAID), in the treatment of Rheumatoid Arthritis in a double-blind, randomized controlled trial
Source: BMC Musculoskelet Disord. 2016 Aug 8;17:331. doi: 10.1186/s12891-016-1170-0 (PMC4977639; doi:10.1186/s12891-016-1170-0)
Supplement: Additional file 1: — List of Ethics Committees. (DOCX 38 kb) [file 12891_2016_1170_MOESM1_ESM.docx]

**List of Ethics Committees**

Hospital Privado Centro Medico

Naciones Unidas 346

Vélez Sarsfield, Córdoba (X5016KEH)

Instituto CAICI SRL.

Mendoza 2612

Rosario, Santa Fe (S2000PBJ)

Organización Médica de Investigación (OMI)

Uruguay 725 PB, Ciudad Autónoma de Bs As (C1015ABO)

Argentina / Tel +54 11 4372 0308

C.E.R. Centro de Rehabilitación

Laprida 568 (este). San Juan, San Juan (J5402DIL)

Comité de Ética en Investigación Hospital Sirio-Libanes

Campana 4658, Capital Federal

Instituto Reumatológico Strusberg

Av. Emilio Olmos 247 1°Piso (X500EDC), Córdoba, Cordoba Argentina / Tel + 54 351

423 205

CECIC Ethics Committee

Vicente Lopez 1441 (B1878DVB), Quilmes, Buenos Aires / Tel. 5411 4224-6854

AIR- Comité de Ética en Investigación.

Av Cabildo 1131. Piso 4. Dto 11. CP1426AAL .Buenos Aires. Argentina/Tel: (54 11)

4781-2499 / e-mail: [comite_air@yahoo.com.ar](mailto:comite_air@yahoo.com.ar)

Comité Independiente de Ética para ensayos en farmacología Clínica

Av. J.E. Uriburu 774 1°Piso (C1027AAP), Buenos Aires

TE/FAX: 5411 4952-3892

Comité Independiente de Ética para ensayos en farmacología Clínica

Av. J.E. Uriburu 774 1°Piso (C1027AAP), Buenos Aires

TE/FAX: 5411 4952-3892

Comité de ética en investigación DIM Clínica Privada

Espora 18, B1704FAB Ramos Mejía, Buenos Aires

TEL: 5411 5554-8888

CIEM-NOA: Comité Independiente de Ética Medica del Noroeste Arg

Las piedras 496 4°Piso, San Miguel de Tucumán, Tucumán

Comité Independiente de Ética para ensayos en farmacología Clínica

Av. J.E. Uriburu 774 1°Piso (C1027AAP), Buenos Aires

TE/FAX: 5411 4952-3892

Comité de Ética en Investigación Clínica (CEIC)

Larrea 1381 3° A (C1117ABK), Capital Federal

Tel: 5411 4826 3962 / [comitedeetica@fibertel.com.ar](mailto:comitedeetica@fibertel.com.ar)

Comité Independiente de Ética para ensayos en farmacología Clínica

Av. J.E. Uriburu 774 1°Piso (C1027AAP), Buenos Aires

TE/FAX: 5411 4952-3892

Ethics Committee of the City of Vienna

1030 Vienna, Thomas-Klestil-Platz 8, Town Town 1st Floor, CB 12.103

Access: 1030 Vienna, Schnirchgasse 12, Staircase 2, CB 12.103

Schulman Associates IRB

4290 Glendale-Milford Rd, Cincinnati, Ohio (45242)

Biomedical Research Ethics Committee médico Julián Coronel

Cra 59 N° 1E-21, Cali, Valle, Colombia. PBX:6800202

Fernando Chalem Rheumatology Institute Foundation

Calle 73 No. 20A - 27 - Te: 346 6077 Fax: 255 8381 Bogotá, D.C.

CEI Servimed E.U (Ethics committee)

Calle 51 N° 34-17 consultorios 208 C.C. Cabecera, Bucaramanga, Colombia. Te:

6432426. [ceiservimedeu@hotmail.com](mailto:ceiservimedeu@hotmail.com)

The Research Ethics Committee - Riesgo de Fractura

Carrera 13 No.97- 25 - Bogotá, TEL: 6446800 - 5922991 ext. 502 .

El Oriente Research Ethics Committee

Calle 53 # 34 - 20 – Cabecera, Bucaramanga – Colombia

Telephones: (57) 300 618 4784

The Research Ethics Committee - Riesgo de Fractura

Carrera 13 No.97- 25 - Bogotá, TEL: 6446800 - 5922991 ext. 502 .

Research Ethics Committee in the Health Area of the Universidad

del Norte. Km. 5 vía a Puerta Colombia • Apartados Aéreos 1569 -51820 • PBX

Switchboard: 3509509 • Fax: (95) 3598852 • Barranquilla, Colombia

Ethics Committee for Multi-Centric Clinical Trial of the University Hospital Motol

V úvalu 84, 150 06 Praha 5. Tel 224 431 195

Local Ethics Committee of the Hospital in Chomutov

Kochova 1185, 43012 Chomutov Krajská zdravotní a.s., Nemocnice Chomutov o.z.

ZUGUEME Independent Ethics Committee

3a calle 11-36 Zona 15, Colonia, Tecun Uman, Guatemala 01015

TEL(502) 2369 1885 - [info@zugueme.org.gt](mailto:info@zugueme.org.gt)

Medisiininen eettinen toimikunta, HUS

Biomedicum Helsinki 2 C Tukholmankatu 8 C, 7 krs.

PL 705. 00029 HUS. Finland

Ethics Committee of the Medical Faculty of the University of Leipzig

Institute of Clinical Pharmacology, Härtelstraße 16-18, 04107 Leipzig

KIMS Institutional Ethics Committee

1-8-31/1, Minister Road, Secunderabad-500 003, A.P., India

info@kims.co.in. 91 040 4488 5000

Institutional Ethics Committee Institute of Post Graduate Medical Education and

Research

244, A.J.C. Bose Road, Kolkata-20

Bhagwan Mahavir Medical Research Centre

10-1-1, Bhagwen Mahavir Marg, A.C. Guards, Hyderabad – 500 004, A.P. India

Hyderabad Central Ethics Committee

#12-13-392. Street N°1. Lane No 4. Tarnaka. Hyderabad-500017. Andhra Pradesesh,

India. [hyderabadcec@gmail.com](mailto:hyderabadcec@gmail.com)

Ethics Committee – Shalby Hospitals

Opp. Karnavati Club, Sarkej Gandhingar Highway, Ahmedabad 38001. [info@shalby.org](mailto:info@shalby.org)

M.S. Ramaiah Medical College and Teaching Hospital Ethical Review Board

MSR Nagar, Msrit Post, Bangalore 560 054, India

Lithuanian Bioethics Committee

State budget institution, Didzioj Str. 22. LT-01128 Vilnius, Lithuania

ibek@sam.lt. (+370 5) 212 4565

Ethics Committee of Instituto Jalisciense de Investigación Clinica S.A

Penitenciaria No. 20, Col. Centro, Guadalajara, Jalisco.

C.P. 44100, Telephone 38 25 64 93

Committee for Ethics in Research of Centro de Estudios de Investigación Clínica

Especializada. Tlatetilpa #24, Col. Barrio San Lucas, Mexico D.F.. C.P. 04030. Tel/Fax:

(33) 53364206

Bioethics Committee at Investigación Clínica S.A.

Puebla No. 422 -4, Col. Roma, 06700 Mexico D.F. Tel: 5256-4910

Independent Committee for Ethics and Research of Centro de Estudios de Investigación

Básica y Clínica S.C.

Justo Sierra 2821-4, Colonia Vallarta Norte, Guadalajara, Jal. Mexico CP. 44690 Tel:

(33) 36300946

Ethics Committee Hospital de Jesús

Av. 20 de Noviembre 82, C.P. 06090 Mexico D.F.

Hospital Civil Fray Antonio Alcalde Committee for Teaching, Research and Ethics

Hospital no. 278 S.H. C.P.44280, Guadalajara,Jal. <TEL:3614-5501>

Ethics and Research Committee Dr Santiago Ramón y Cajal General State Hospital

ISSTE

Prol. Predio Canoas S/N, Silvestre Dorador, 34070 Victoria De Durango, DGO, México.

+52 618 825 1318

Health Research Ethics Committees (CEIS) of the Clinical Research Unit of the Centro

de Especialidades Médicas del Sureste S.A. de C.V

Calle 60 No.329-B entre 35 y Av.Colón, C.P. 97000. Mérida, Yucatán.

Comité de Bioética de la investigación de Instituto Conmemorativo Gorgas de estudios

de la Salud

Ave. Justo Arosemena y Calle 35 Tel.: (507) 527-4811• Apartado Postal N° 0816-02593,

Panamá.

Comité Institucional de Ética en Investigación de la Universidad de San Martin de Porres.

Av. Las Calandrias s/n Santa Anita, Lima - Perú, Tel: (511) 362-0064.

ONGD Asociación Benéfica Prisma.

Calle Carlos Gonzales 251 Urb. Maranga. Lima 32, Perú. (170070).

Tel. (511) 6165500. [prisma@prisma.org.pe](mailto:prisma@prisma.org.pe)

Komisja Bioetyczna przy Okregowej

Izbie Lekarskiej w Bialymstoku

(Bioethics Committee at the Regional Medical Chamber of Physicians and Dentists in

Bialystok) Ul. Swietojanska 7. 15-082 Bialystok

Komisja Bioetyczna przy Okregowej

Izbie Lekarskiej w Gdansku (Bioethics

Committee at the Regional Chamber of Physicians and Dentists in

Gdansk) Ul. Sniadeckich 33. 80-204 Gdansk

Bioethics Committee at the Regional Medical Council of Regional Chamber of

Physicians and Dentists in Poznan

61-734 Pozna􀄔, ul. Nowowiejskiego 51. Tel. (0-61) 852-58-60, Fax.: (061) 851-87-62

Bioethics Committee at the Regional Medical

Council of Regional Chamber of Physicians and Dentists in Poznan

61-734 Pozna􀄔, ul. Nowowiejskiego 51

Tel. (0-61) 852-58-60, Fax.: (061) 851-87-62

Komisja Bioetyczna przy Okregowej Radzie Lekarskiej Wielkopolskiej Izby Lekarskiej

(Bioethics Committee at the Regional Medical Council of the Regional Chamber of

Physicians and Dentists in Poznan)

Ul. Nowowiejskiego 51. 61-734 Poznan

Bioethics Committee at the Regional Medical

Council of Regional Chamber of Physicians and Dentists in Pozna􀄔

61-734 Poznan, ul. Nowowiejskiego 51

Tel. (0-61) 852-58-60, Fax.: (061) 851-87-62

Komisja Bioetyczna przy Okr􀄊gowej

Izbie Lekarskiej w Krakowie (Bioethics Committee at the Regional

Chamber of Physicians and Dentists in Cracow)

Ul. Krupnicza 11a

31-123 Krak6w

Bioethics Committee at the Regional Medical

Council of

Regional Chamber of Physicians and Dentists in Poznan

61-734 Poznan, ul. Nowowiejskiego 51

Tel. (0-61) 852-58-60, Fax.: (061) 851-87-62

Komisja Bioetyczna przy Okr􀄊gowej

Izbie Lekarskiej w Warszawie

(Bioethics Committee at the Regional

Chamber of Physicians and Dentists

in Warsaw)

Ul. Pulawska 18

02-512 Warszawa

Ministry of Health. National Ethics Committee for Clinical Study of Medicines.

011478 Bucharest, 48 Av. Sanatescu Street, district 1

Phone: 0314051076; Fax: 0314051075

State Budgetary Educational Institution of Higher Professional Education

KEMEROVO STATE MEDICAL ACADEMY FEDERAL SERVICE ON

SURVEILLANCE IN HEALTHCARE AND SOCIAL DEVELOPMENT

22a Voroshylova ul., Kemerovo, 650029

tel. (8-3842) – 734856, fax (8-3842) – 734856

GBKUZ of Yaroslavl region “Cit

7, Semashko ul. Yaroslavl Yaroslavskaya oblast' 150002 RUSSIAN FEDERATION / Tel

+79038240506

GOU VPO Novosibirsk State Med

52, Krasniy prospect Novosibirsk Novosibirskaya oblast' 630091 RUSSIAN

FEDERATION / Tel +7 913 901 82 82

Clinical hospital n.a.N.V.Solo

11 Zagorodny Sad str Yaroslavl Yaroslavskaya oblast' 150003 RUSSIAN

FEDERATION / Tel +7 4852 73 71 03

KIMI-LEC

197110, St Petersburg, Krestovskiy pr., 18, tel. +7 921 930 9984

State Healthcare Institution Regional Clinical Hospital

1 Smirnovskoe Gorge, Saratov,Russia, 1410053

Tel.: +7 8452 491467

Independent Ethics Committee of Smolensk State Medical Academy

28 ul. Krupskoj, 214019, Smolensk, Russia

Rheumatology Consultation and Diagnostics Center “Healthy Joints”

33 Romanov St., Novosibirsk, Russia, 630091

Tel.: +7 (383) 33100 49; Fax: +7 (383) 222-54-03

e-mail: info@zsustav.ru; Web-site: [www.zsustav.ru](http://www.zsustav.ru)

Ethics Committee at Gosudarstvennoye byudzhetnoye uchrezhdeniye zdravookhraneniya

[State Budgetary Healthcare Institution] of the Republic of Karelia.

Medical Institute for Scientific Research «Your Health» LLC

2 Dostoyevskiy St., Kazan, Russia, 420097

Tel./Fax: (843) 537 93 93

State Healthcare Institution of the City of Moscow Municipal Clinical Hospital No. 1 n.a.

N.I. Pirogov

8 Leninskiy Ave., Moscow, Russia, 119049

Tel.: (495)2366096, Fax: (495)2366528

Urad Trnavskeho Samospra Vneho Kraja

Eticka Komisia. P.O BOX 128, Strarohajska 10

917 01 Trnava

National Institute of Rheumatic Diseases

Nábr. I. Krasku 4, 921 12 Piešt’any, Slovak Republic

Pharma-Ethics Independent Research Ethics Committee

123 Amcor Road, Lyttelton Manor, 0157

Institutional Review Board Chang Gung Medical Foundation

No. 199, Tung Hwa North Road, Taipei City 105 . Fax: 03-3494549

Contact Person & Phone: Ting-Yi Wu; 03-3196200 ext. 3704

Taipei Veterans General Hospital, Vac

201 Shih-pai road, Sec 2. Taipei, Taiwan. Republic of China

China Medical University Hospital IRB

No. 2 Yuh Der Road, Taichung Taiwan R.O.C

Joint Institutional Review Board

No.5-1 Lane 331, Sec 2, Shih-Pai Road, Taipei (11217), Taiwan. R.O.C

Institutional Review Board Committee, Changhua Christian Hospital

135, Nan-Hsiao Street, Changhua 500, Taiwan

Buddhist Tzu Chi General Hospital Research e Ethics Committee

707, Sec.3, Chung-Yang Rd., Hualien, 97002, Taiwan, R.O.C.

Institutional Review Board Chung Shan Medical University Hospital

No. 110, Sec.1, Chien-Kuo N. Road, Taichung, Taiwan 402, R.O.C

Research Ethics Committee National Taiwan University Hospital

7, Ching-Shan South Road, Taipei, Taiwan 100, R.O.C

National Research Ethics Services Cambridgeshire 2 Research Ethics Committee.

Victoria House, Capital Park, Fulbourn, Cambridge CB21 5XB

Schulman Associates IRB, Inc

4445 Lake Forest Drive Suite 300, Cincinnati Ohio 45242 UNITED STATES / Tel 888-

557-2472 / [dbattson@sairb.com](mailto:dbattson@sairb.com)

**List of Investigators**

Country - Investigator

Argentina - Alvarellos,Alejandro

Argentina - Machado, Daniel Agusto

Argentina - Mysler,Eduardo

Argentina -PardoHidalgo,Rodolfo

Argentina - Rillo,Oscar

Argentina - Strusberg,Ingrid

Argentina - Velasco, Jorge

Argentina - Venarotti,Horacio Oscar

Argentina - Berman,Alberto

Argentina - Ruggieri,Lucia

Argentina - Capozzi,Maria

Argentina - Lucero,Eleonora

Argentina - Leal,Maria

Argentina - Ariel,Federico

Argentina - Cavallasca,Javier

Austria - Cauza,Edmund

Austria - Erlacher,Ludwig

Austria - Groblschegg,Susanne

Canada - Choquette,Denis

Canada - McCarthy,Timothy

Canada - Rodrigues,Jude

Colombia - Coronel,Julian

Colombia - Mendez,Paul

Colombia - Otero,William

Colombia - Diaz,Mario

Colombia - Saaibi,DiegoLuis

Colombia - Velez,PatriciaJulieta

Colombia - Londono,John

Colombia - Forero,Elias

Czech Republic - Bortlik,Ladislav

Czech Republic - Jonas,Petr

Czech Republic - Mosterova,Zdenka

Czech Republic - Nemec,Petr

Czech Republic - Skorepova,Iva

Czech Republic - Vitek,Petr

Czech Republic - Urbanova,Zuzana

Finland - Paimela,Leena

Germany - Alten,Rieke

Germany - Baerwald,Christoph

Germany - Berge,Hartwin

Germany - Blaschke,Sabine

Germany - Contzen,Christel

Germany - Degtyareva,Elizaveta

Germany - Haux,Roland

Germany - Meissner,Gudrun

Germany - Mueller-Ladner,Ulf

Germany - Rinke,Andrea

Germany - Piechatzek,Richard

Germany - Wassenberg,Siegfried

Germany - Nischik,Ruth

Germany - Schenkenberger,Isabelle

Germany - Lahne,Uwe

Germany - Spieler,Wolfgang

Guatemala - Briones,Henry

Guatemala - Kutzbach,Abraham NAP 10-Apr-13 28-Nov-13 None

Guatemala - Chavez,Nilmo

Guatemala - HerreraMendez,Maynor

Guatemala - Rivera,Ruddy

Guatemala - TunaCastro,Maria

Guatemala - RosalPalomo,Erick

India - ChandraMouli,Sarath

India - Gosh,Alokendu

India - KishoreKadel,Jugal

India - Pal,Sarvajeet

India - Sharma,Reena

India - Shetty,Naresh

Lithuania - Milasiene,Roma

Lithuania - Kriauciuniene,Virginija

Lithuania - Eidziuniene,Jurate

Lithuania - Pociene,Gilvina

Mexico - Avila Armengol,Hilario Ernesto

Mexico - Gonzaga,Jaime Reyes

Mexico - Garcia,Conrado

Mexico - GarciadelaTorre,Ignacio

Mexico - Olguín Ortega,Maria de Lourdes

Mexico - Gutiérrez Ureña,Sergio Ramon

Mexico - Ortiz Jimenez,Enrique

Mexico - Simon Campos,Jesus

Panama - Guerra,Generoso

Peru - PazGastanaga,Maria

Peru - BellatinVargas,Luis

Peru - Gomez,Sonia

Peru - Felix,Romero

Peru - Portocarrero,Gustavo

Peru - Alfaro,Jose

Peru - Perich,Risto

Poland - Badurski,Janusz

Poland - Bazela,Barbara

Poland - Dudek,Anna

Poland - Glogowska-Szelag,Joanna

Poland - Grabowicz-Wasko,Barbara

Poland - Hrycaj,Pawel

Poland - Mazurek,Marcin

Poland - Olas,Jacek

Poland - Porawska,Wieslawa

Poland - Racewicz,Artur

Poland - Ruzga,Zofia

Poland - Szymanska,Malgorzata

Poland - Dudek,Anna

Poland - Guzera,Zbigniew

Poland - Stanislawski,Piotr

Romania - BIAGINI,MirelaIuliana

Romania - CODREANU,Catalin

Romania - MOCIRAN,Eugenia

Romania - PAVEL,Mariana

Romania - RADULESCU,Florin

Romania - REDNIC,Simona

Romania - NEDELCIU,Malina

Romania - UDREA,Gabriela

Romania - REZUS,Elena

Romania - Georgescu,EmiliaMaria

Romania - Cristei, Dorica

Romania - CHIRIEAC,RodicaMarieta

Romania - PETRE,Octavian

Russia - Barbarash,Olga

Russia - Chizhov,Petr

Russia - Demin,Alexander

Russia - Ershova,Olga

Russia - Makulova,Tatiana

Russia - Reshetko,Olga

Russia - Zharkova,Liudmila

Russia - Zonova,Elena

Russia - Vezikova,Natalia

Russia - Myasoutova,Leisan

Russia - Shmidt,Evgeniya

Slovakia - Durisova,Elena

Slovakia - Letkovska,Alexandra

South Africa - Louw,Ingrid

South Africa - Nel,Debra

South Africa - Spargo,CathyE

South Africa - JansenvanRensburg,Barend

South Africa - Matsiliza,Noma

South Africa - Reuter,Helmuth

Taiwan - Chen,Ying-Chou

Taiwan - Chou,Chung-Tei

Taiwan - Huang,Chung-Ming

Taiwan - Lu,Ling-Ying

Taiwan - Su,Che-Chun

Taiwan - Tsai,Shih-Tzu

Taiwan - Wei,Cheng-Chung

Taiwan - Yu,Chia-Li

United Kingdom - George,Emmanuel

United Kingdom - Walker,David

United Kingdom - Balaji,Hiremagalur

United Kingdom - Richards,Selwyn

United Kingdom - Woolf,Anthony

United Kingdom - Fairburn,Kevin

United Kingdom - Salih,Abdelrazig

United States - Ansari,Saadat

United States - Bennett,Ralph

United States - Birbara,Charles

United States - Bookbinder,Stephen

United States - Busch,Howard

United States - Lakanpal,Sharad

United States - Portnoy,Edward

United States - Diri,Erdal

United States - Fenton,Ira

United States - Fiechtner,Justus

United States - Franklin,C.Michael

United States - Goddard,David

United States - Goldman,John

United States - Hartman,Sanford

United States - Hole,Susan

United States - Holt,PeterA.

United States - Hou,Antony

United States - Hsu,Vivien

United States - Ignaczak,Thomas

United States - Kirby,Daniel

United States - Kivitz,Alan

United States - Kumar,Usharani

United States - Lovell,Tracy

United States - Majjhoo,Amar

United States - Makarowski,William

United States - Mandel,David

United States - Mathews,Steven

United States - McAdam,Lawrence

United States - Mcllwain,Harris

United States - Neuwelt,C.Michael

United States - Porges,Andrew

United States - Prupas,Malin

United States - Saadeh,Constantine

United States - Schimizzi,Gregory

United States - Sebba,Anthony

United States - Sherrer,Yvonne

United States - Shlotzhauer,Tammi

United States - Lawson,Jeffrey

United States - Sunkureddi,Prashanth

United States - Valenzuela,Guillermo

United States - Waller,Philip

United States - Wiesenhutter,Craig

United States - Lane,Nancy

United States - Olvera,Noe

United States - Shergy,William

United States - Hull,John

United States - Mehta,Daksha

United States - Ftetzin,Scott

United States - Sisay,Moges

United States - Songcharoen,Suthin

United States - Willette,Roger

United States - Gimness,Michael

United States - Koilpillai,Robinson

United States - Jacobs,Shahram

United States - St. John,Melody

United States - Griffin,Robert

United States - Raikhel,Marina

United States - Kirstein,Judith

United States - Rogers,Anthony

United States - Paul,Donna

United States - Neal,Jeffrey

United States - Snyder,Arthur
