# Supplementary material for: Evaluation of two doses of etoricoxib, a COX-2 selective non-steroidal anti-inflammatory drug (NSAID), in the treatment of Rheumatoid Arthritis in a double-blind, randomized controlled trial
Source: BMC Musculoskelet Disord. 2016 Aug 8;17:331. doi: 10.1186/s12891-016-1170-0 (PMC4977639; doi:10.1186/s12891-016-1170-0)
Supplement: Additional file 2: Table S1. — Summary of Additional Endpoints during Part I (6 weeks of treatment). Table S2. Summary of Additional Endpoints during Part II Among Inadequate Responders from Part I. (DOCX 21 kb) [file 12891_2016_1170_MOESM2_ESM.docx]

**Supplement 2**

Table S1 - Summary of Additional Endpoints during Part I (6 weeks of treatment)

|  | Placebo | Etoricoxib 60 mg | *LS Mean Difference vs. Placebo; p-Value*‖ | Etoricoxib 90 mg | *LS Mean Difference vs. Placebo; p-Value* | LS Mean Difference Between 60 mg and 90 mg; p-Value‖ |
| --- | --- | --- | --- | --- | --- | --- |
| LS mean change from baseline Tender Joint Count (Total 28 Joints) | 5.97 (-6.97, -4.98 | - 7.56 (-8.03, -7.08) | *-1.58 (-2.60, -0.57)* | - 7.35 (-7.93, -6.78) | *-1.38 (-2.44, -0.32)* | *0.20 (-0.39, 0.80)* |
| LS mean change from baseline Tender Joint Count (Total 68 Joints) | -10 .48 (-12.24, -8.71) | - 13.01 (-13.85, -12.18) | *-2.53 (-4.33, -0.74)* | - 1 2 .91 (-13.93, -11.90) | *-2.43 (-4.32, -0.55)* | *0.10 (-0.96, 1.16)* |
| LS mean change from baseline Swollen Joint Count (Total 28 Joints) | - 4.97 (-5.79, -4.15 | - 5.78 (-6.17, -5.39) | *-0.81 (-1.64, 0.02)* | - 5.78 (-6.25, -5.32) | *-0.81 (-1.69, 0.06)* | *-0.00 (-0.49, 0.49)* |
| LS mean change from baseline Swollen Joint Count (Total 68 Joints) | - 6.83 (-8.04, -5.61) | - 8.39 (-8.97, -7.81) | *-1.57 (-2.81, -0.33)* | 8.39 (-9.09, -7.70) | *-1.57 (-2.86, -0.27)* | *-0.00 (-0.73, 0.73)* |
| LS mean change from baseline Patient Global Assessment of Disease Activity | 14.01 (-17.70, -10.33) | -22.78 (-24.54, -21.02) | *-8.77 (-12.53, -5.01)* | -25.51 (-27.63, -23.39) | *-11.50 (-15.43, -7.56)* | *-2.73 (-4.94, -0.52)* |
| LS mean change from baseline Investigator Global Assessment of Disease Activity | - 0.84 (-0.98, -0.70) | - 1.12 (-1.18, -1.05) | *-0.28 (-0.42, -0.14)* | - 1.13 (-1.21, -1.05) | *-0.29 (-0.44, -0.14)* | *-0.01 (-0.09, 0.08)* |
| LS mean change from baseline Health Assessment Questionnaire | - 0.20 (-0.27, -0.12) | - 0.33 (-0.37, -0.30) | *-0.14 (-0.21, -0.06)* | - 0.36 (-0.40, -0.32) | *-0.17 (-0.25, -0.09)* | *-0.03 (-0.07, 0.02)* |
| LS geometric mean ratio (on treatment/baseline) C-Reactive Protein | 0.94 (0.80, 1.09) | 0.87 (0.81, 0.93) | *0.93 (0.79, 1.08)* | 0.89 (0.82, 0.97) | *0.95 (0.81, 1.12)* | *1.03 (0.94, 1.12)* |

‖ Comparisons for C-Reactive Protein show between- LS geometric mean ratio (95%CI)

Table S2: Summary of Additional Endpoints during Part II Among Inadequate Responders from Part I

|  | Etoricoxib 60 mg (Part I) / 60 mg (Part II) | Etoricoxib 60 mg (Part I) / 90 mg (Part II) |
| --- | --- | --- |
| LS Mean Change from Week 6 in Tender Joint Count (Total 28 Joints)Over Weeks 10 and 12 | 1.64 (-2.48, -0.80) | -1.37 (-2.19, -0.55) |
| LS Mean Change from Week 6 in Tender Joint Count (Total 68 Joints)Over Weeks 10 and 12 | -3.34 (-4.70, -1.99) | -2.43 (-3.77, -1.09) |
| LS Mean Change from Week 6 in Swollen Joint Count (Total 28 Joints)Over Weeks 10 and 12 | -0.59 (-1.25, 0.08) | -0.98 (-1.64, -0.33) |
| LS Mean Change from Week 6 in Swollen Joint Count (Total 66 Joints)Over Weeks 10 and 12 | -0.73 (-1.65, 0.19) | -1.03 (-1.94, -0.12) |
| LS Mean Change from Week 6 in Patient Global Assessment of Disease Activity Over Weeks 10 and 12 | -8.67 (-11.67, -5.68) | -8.87 (-11.83, -5.92) |
| LS Mean Change from Week 6 in Investigator Global Assessment of Disease Over Weeks 10 and 12 | -0.13 (-0.24, -0.02) | -0.11 (-0.22, -0.01) |
| LS Mean Change from Week 6 in Health Assessment Questionnaire Over Weeks 10 and 12 | -0.09 (-0.14, -0.03) | -0.02 (-0.07, 0.04) |
| LS Geometric Mean Ratio from Week 6 C-Reactive Protein Over Weeks 10 and 12 | 1.03 (0.90, 1.18) | 1.04 (0.91, 1.19) |
